# Interaction of single- and double-stranded DNA with multilayer MXene by fluorescence spectroscopy and molecular dynamics simulations†
†Electronic supplementary information (ESI) available. See DOI: 10.1039/c9sc03049b


**DOI:** 10.1039/c9sc03049b

**Published:** 2019-09-23

**Authors:** C. Lorena Manzanares-Palenzuela, Amir M. Pourrahimi, J. Gonzalez-Julian, Zdenek Sofer, Martin Pykal, Michal Otyepka, Martin Pumera

**Affiliations:** a Center for Advanced Functional Nanorobots , Department of Inorganic Chemistry , University of Chemistry and Technology Prague , Technická 5 , Prague 6 , 166 28 , Czech Republic . Email: martin.pumera@vscht.cz; b Forschungszentrum Jülich GmbH , Institute of Energy and Climate Research, Materials Synthesis and Processing (IEK-1) , 52425 Jülich , Germany; c Regional Centre for Advanced Technologies and Materials , Palacký University Olomouc , Šlechtitelů 27 , Olomouc , 771 46 , Czech Republic; d Future Energy and Innovation Laboratory , Central European Institute of Technology , Brno University of Technology , Purkyňova 656/123 , Brno , CZ-616 00 , Czech Republic; e Department of Chemical and Biomolecular Engineering , Yonsei University , 50 Yonsei-ro, Seodaemun-gu , Seoul 03722 , Korea

## Abstract

The integration of nucleic acids with nanomaterials has attracted great attention from various research communities in search of new nanoscale tools for a range of applications, from electronics to biomedical uses. MXenes are a new class of multielement 2D materials baring exciting properties mostly directed to energy-related fields. These advanced materials are now beginning to enter the biomedical field given their biocompatibility, hydrophilicity and near-infrared absorption. Herein, we elucidate the interaction of MXene Ti_3_C_2_T_*x*_ with fluorophore-tagged DNA by fluorescence measurements and molecular dynamics simulations. The system showed potential for biosensing with unequivocal detection at picomole levels and single-base discrimination. We found that this material possesses a kinetically unique entrapment/release behavior, with potential implications in time-controlled biomolecule delivery. Our findings present MXenes as platforms for binding nucleic acids, contributing to their potential for hybridization-based biosensing and related bio-applications.

## Abstract 2

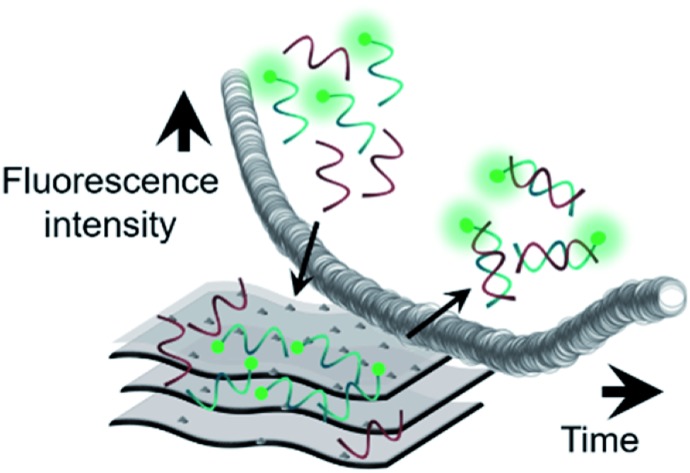
MXenes show differential affinity towards single- and double-stranded DNA, with unique kinetics and potential for fluorescent biosensing.

## Introduction

The interaction of nucleic acids with micro/nanomaterials has been an extensively studied topic with relevant implications in different fields. In materials science, DNA has been utilized as “biological glue” for achieving programmable and precise assembly at the nanoscale. The resulting DNA-linked structures can generate highly ordered nanoparticle structures with possibility of modulation of their optical, magnetic, and electronic properties.^1^–^3^ In molecular biology and bioanalytical chemistry, the adsorptive properties of some materials towards nucleic acids have been exploited for the extraction of large and small DNA fragments from biological fluids in sample cleanup.^4^,^5^ DNA physisorption onto 2D materials, for example, has been broadly applied in biosensing and nanopore-based sequencing.^6^–^9^ Material-based biosensing systems have become very popular over the past decade.^10^–^12^ They rely on fluorescence resonance energy transfer (FRET), taking advantage of the differential adsorption affinity towards single- and double-stranded DNA (ssDNA and dsDNA, respectively). The detection principle consists of fluorescence quenching upon ssDNA adsorption and fluorescence recovery after duplex formation. This enables homogeneous hybridization-based detection assays, which are easy and simple to operate while retaining nanomolar limits of detection and high sensitivity.^13^ Although biosensing might just be the most popular application of DNA–material interactions, the role of such basic research in biomedical and bionanotechnology-related applications is also extensive. A few of them include targeted drug and gene delivery, imaging theranostics, phototherapy, *in vivo* biosensing, and tissue engineering.^14^–^19^ The appeal of 2D layered materials is based on their high aspect ratio and unique structural and electronic properties, offering a great degree of tuneability. Their characteristic interaction with nucleic acids has driven researchers to continue looking into new layered materials for capturing, analyzing and delivering of these biomolecules.^20^,^21^

**Fig. 1:**
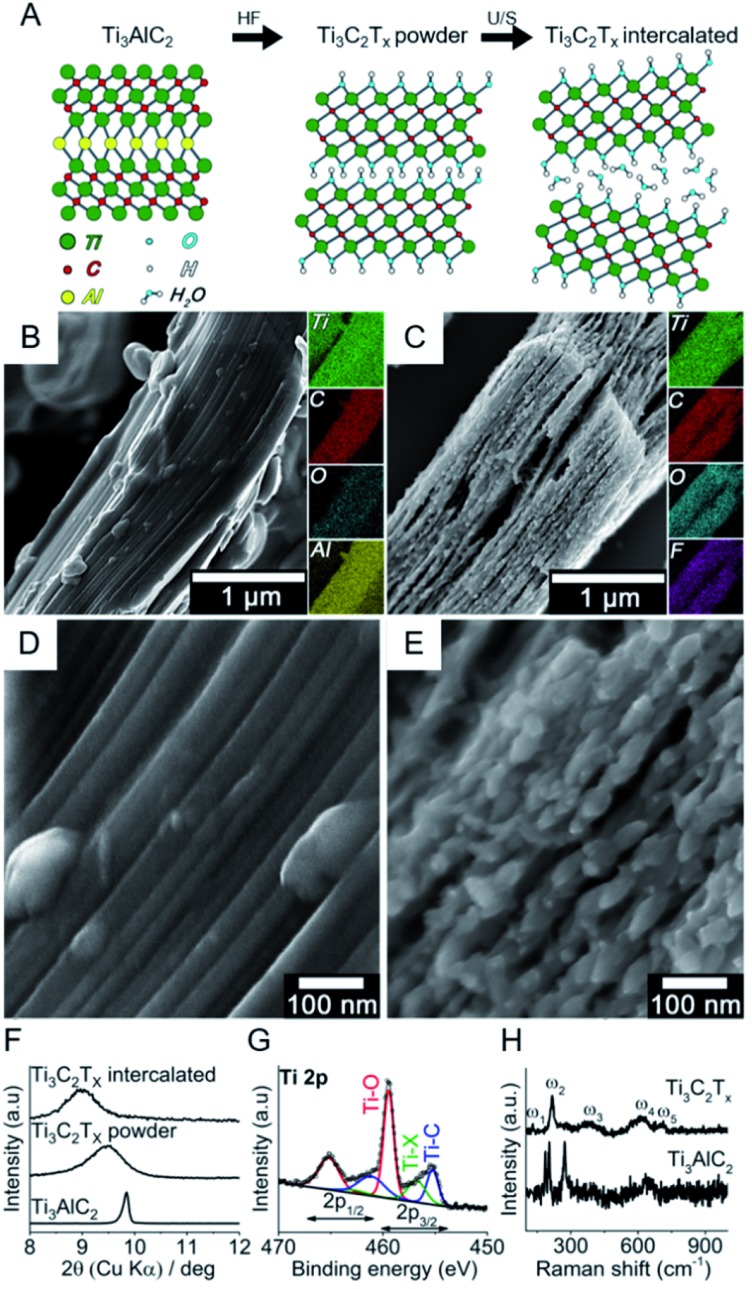
Synthesis and characterization of intercalated MXene: (A) schematic etching (HF) and delamination (*via* ultrasound, U/S) processes; (B and C) representative SEM images together with EDS elemental mapping for MAX precursor and intercalated MXene; (D and E) high-resolution SEM images for MAX precursor and intercalated MXene; (F) X-ray diffractograms; (G) high-resolution XPS of the Ti 2p region for intercalated MXene; and (H) Raman spectra of the MAX precursor and intercalated MXene.

MXenes represent a new family of 2D materials consisting of transition metal carbides and carbonitrides first introduced in 2011 by Naguib *et al.*^22^ The authors presented this new family as complex, layered structures that offer a wide range of properties owed to their multielement content and tunable composition. As shown in Fig. 1A, they are prepared from the respective MAX phase by selectively etching an A-group element (*e.g.* aluminium) generating MXenes with terminal groups (–OH, –F) that render hydrophilic surfaces. The resulting etched structures exhibit accordion-like shapes with interlayer spaces that can serve as molecular sieving channels and for hosting ions and organic molecules.^23^–^25^ Further delamination can be achieved with different methods, resulting in ultrathin 2D sheets.^26^ The applications include but are not limited to: environmental remediation, photocatalysis, electromagnetic shielding, sensing and energy storage.^27^–^31^ Recently, MXenes started to be considered as promising candidates for bio-applications mainly due to their hydrophilicity and biocompatibility, plus their strong absorbance in the near-infrared region and adsorptive properties.^32^–^35^ Very recently, a nanopore DNA sequencing system has been reported with MXene membranes.^36^ Aptamer-based assays have also been recently developed for exosome detection,^37^,^38^ as well as MXene composites with DNA for dopamine detection.^39^ Herein, we probe the interaction between single- and double-stranded DNA with Ti_3_C_2_T_*x*_, the most studied and widely used MXene, by means of fluorescence spectroscopy and molecular dynamics (MD) simulations. We aim at investigating such interaction at a basic level to explore the capabilities of this kind of materials not only as prospective biosensing platforms for sequence-specific DNA detection, but also as potential carriers of nucleic acids serving as structural support and biomolecular reservoirs for biomedical applications.

## Results and discussion

**Scheme 1:**
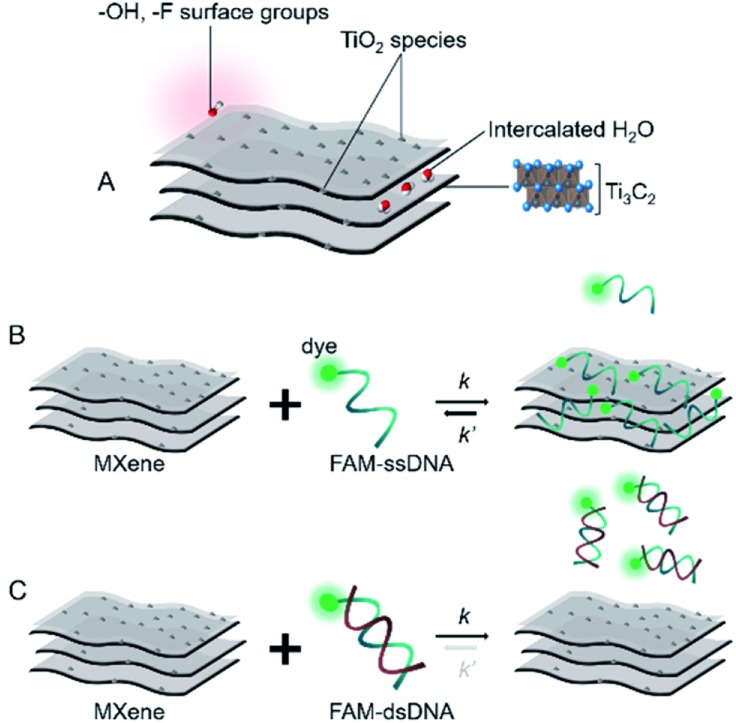
Simplified representation of (A) MXene; (B and C) projected interaction between MXene with ssDNA and dsDNA, respectively.

Based on the already well-established knowledge of DNA–material interaction, we projected that the MXene–DNA interaction (Scheme 1) would be based on different affinities towards ssDNA and dsDNA, the former having higher affinity towards the material than the latter. Noncovalent binding of ssDNA to the surface of nanomaterials is generally based on weak interactions such as van der Waals forces, hydrogen bonds and π stacking, involving the phosphate backbone and/or the nucleobases, respectively. π–π interactions are primarily associated to sp^2^-hybridized systems like graphene, whereas the first two types are more ubiquitous within a wide range of materials. Adsorption of dsDNA to these surfaces is usually much weaker due to the higher rigidity of the double-helix compared to the single-stranded form. If the adsorption is taking place mainly *via* π–π stacking, then it is expected that dsDNA is far less likely to interact once the bases are not free to interact with the material surface.

Fig. 1 shows the bottom-up synthesis of partially delaminated MXene (Ti_3_C_2_T_*x*_) from the MAX phase precursor (Ti_3_AlC_2_), together with the discreet top-down characterizations to exhibit the structure of MXene from micro-scale to molecular level (see Experimental section in ESI†). The MAX precursor contains layers of transition titanium carbides (Ti_3_C_2_), which are interleaved with layers of Al-element atoms (Fig. 1A). In the next step, HF is used to selectively etch the Al-element from MAX phase to achieve MXene phase. The obtained MXene phase has a mixture of –OH, –O and –F terminations, with the chemical formula Ti_3_C_2_T_*x*_, where T represents the surface terminations.^24^,^27^ The further delamination step *via* ultrasonication in aqueous medium results in an intercalated structure, wherein the water molecules can fit in and expand the interlayer spacing and consequently increase the final specific surface area.^27^,^40^,^41^

In order to assess the nano/microstructure of MAX precursor and intercalated MXene, their SEM images coupled with EDS elemental map are shown in Fig. 1B and C, respectively. The EDS results confirmed that the Al element was almost removed from the precursor through the etching process, and the new atomic terminations *i.e.* –OH, –O and –F were introduced to the MXene phase. The high-resolution SEM images show the morphological changes after etching (Fig. 1D and E). The intercalation of the multilayer is evident after the etching process. The small species that appeared on the edges of MXene layers are most likely TiO_2_ nanocrystals, for it is well-known by now that TiO_2_ nanoparticles form on MXene surfaces due to the fast oxidation of titanium carbide in aqueous and/or oxygen conditions and the process starts on the edges.^27^,^42^–^45^ More SEM images of the multilayer flakes together with larger accordion-like structures can be found in Fig. S1 in ESI.† The dispersion was let to sediment and the fine supernatant particles consisting mainly of multilayer flakes were retrieved for further characterization and DNA tests. Fig. S2† shows the atomic force microscopy (AFM) images of these typical layered structures and their flake dimensions with width values ranging from 0.9 to 1.2 μm and overall heights from 100 to 140 nm. The transition electron microscopy (TEM) images show individual multilayer flakes of micron size (Fig. S3A and B†). The high resolution TEM images shows a lattice fringe distance of 0.20 nm, consistent with the (101) crystal plane of Ti_3_C_2_ (ref. 46) (Fig. S3C†). The selected area electron diffraction (SAED) shows the hexagonal symmetry of Ti_3_C_2_ MXene. Individual main diffraction patterns are indexed on the image (Fig. S3D†).

The intercalated MXene was further examined by bulk (XRD) and surface (XPS) characterizations. Fig. 1F shows the X-ray diffractogram of MAX phase together with MXene phases after etching (Ti_3_C_2_T_*x*_ powder) and ultrasonication (Ti_3_C_2_T_*x*_ intercalated) in the small angle (2*θ*) regions, *i.e.* 8–10°, to explore the interlayer spacing. The crystal interlayer distances (*d*) were calculated from the Bragg equation (*λ* = 2*d* sin *θ*), where *λ* and 2*θ* are the wavelength of Cu-Kα source (1.54178 Å) and (002) peak-position as related to *c* lattice parameter of hexagonal close-packed crystal structure.^27^,^40^,^47^ After etching, the (002) peak shifted to smaller angles and broadened, which respectively indicates the increase in interlayer spacing and reduction in crystal size according to Bragg and Scherrer equations.^47^ The ultrasonication of multilayer MXene resulted in a further shift to smaller XRD angles. It is therefore shown that the etching and intercalation with water molecules increase the interlayer spacing to 10% (*i.e.* from 0.9 to 1 nm). Full-range X-ray diffractograms of MAX phase and intercalated MXene are shown in Fig. S4.† The majority of the non-basal plane peaks of Ti_3_AlC_2_ disappeared after HF etching/ultrasonication in water.^48^ The presence of different TiO_2_ phases, *i.e.* anatase and rutile, in T_3_C_2_T_*x*_ was discreetly evidenced in the 25–28° angle (2*θ*) region.^49^,^50^ Fig. 1G shows the high-resolution spectrum of Ti 2p photoelectron region for the intercalated MXene. The chemical composition consists of oxygen-rich moieties (56%), titanium carbide (29%) and other terminal functionalities such as –F groups (15%). The peak separation of 5.6 eV for the Ti 2p_3/2_ and Ti 2p_1/2_ suggests that there is a contribution from TiO_2_ together with Ti–O terminal groups.^51^ The presence of –OH terminations is furthermore confirmed by high-resolution XPS spectra for O 1s region (Fig. S5†), which is essential for the adsorption of polar species.^52^,^53^ It was reported that the water molecules cannot be removed by further drying and ordinary degassing steps,^27^,^40^ where its presence is observed in our case (Fig. S5†). Raman analysis showed the vibrational modes for Ti_3_AlC_2_ and Ti_3_C_2_T_*x*_ MXene obtained after etching (Fig. 1H). The former exhibited vibrational modes at 183, 203 and 273 cm^–1^, which have been assigned to Al atoms. Other modes located between 600 and 700 cm^–1^ have been associated to C atoms and can be seen in both spectra.^54^ As expected, Al-related modes disappear after etching. The modes for Ti_3_C_2_T_*x*_ MXene are located at 128, 219, 385, 618 and 711 cm^–1^, comparable to previous reports.^55^–^57^ The heterogeneity of the surface terminal groups affect the overall spectrum as a result of collaborative vibrations from surface and central Ti atoms, central C atoms, and the terminal groups –O, –OH and –F. The band at 128 cm^–1^ (E_g_) is indicative of –F terminal groups from in-plane vibrations of surface Ti and C atoms. Out-of-plane stretching vibrations of surface Ti and C atoms give rise to the 219 cm^–1^ band as a result of –OH terminal groups. The 380 cm^–1^ hump can be associated with heterogeneously distributed –O and –OH terminations, while both –F and –OH groups contribute to the in-plane vibration of the C atoms at 618 cm^–1^.^55^ The presence of TiO_2_ was not evident with Raman spectroscopy, suggesting that this chemical species is not predominant in the system.

A picture of the stable MXene dispersion is shown in Fig. S6† with its corresponding absorbance spectrum showing characteristic absorption at *ca.* 760–800 nm.^43^ The stability of the final dispersion was also corroborated with zeta potential measurements at pH ∼ 7 (*ζ* = –29.7 ± 7.4 mV). In order to gain more information about the surface area and the pore structure, the BET non-local density functional theory (NLDFT) pore size distributions at 77 K are shown for both MAX precursor and MXene phase in Fig. S7A and B,† respectively. After the etching process, the volume of mesopores was sharply increased, which was already illustrated by SEM local measurement (Fig. 1D and E). The relatively large amount of mesopores show the potential for entrapping small fragments of nucleic acids.

**Fig. 2:**
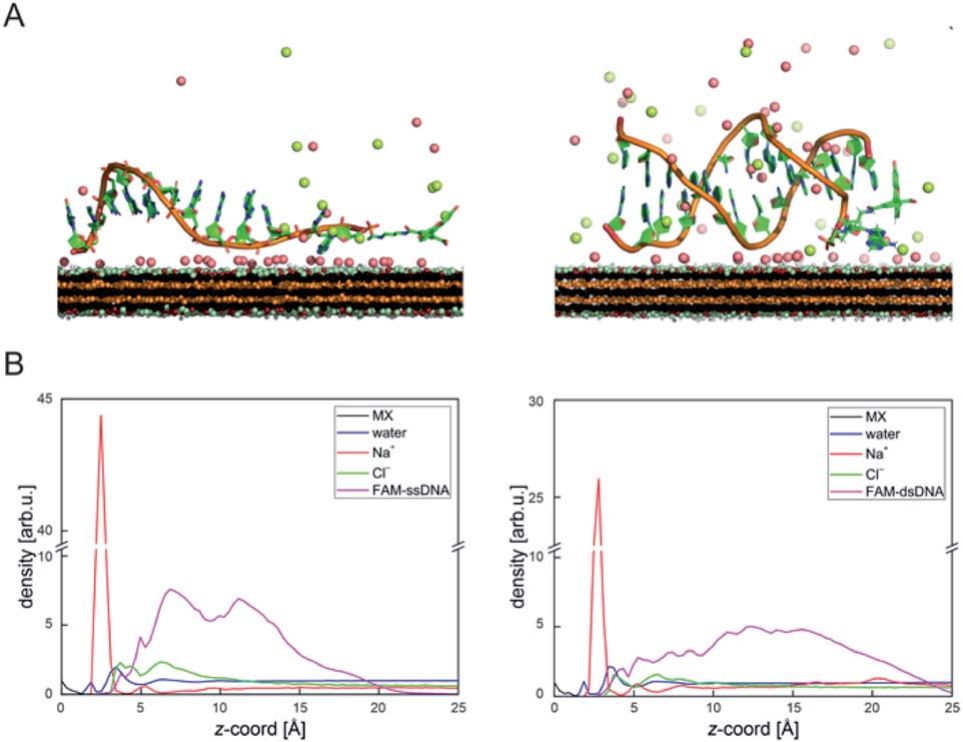
(A) Structures taken from MD simulation showing the typical binding pattern of FAM-ssDNA (left) and FAM-dsDNA (right) on MXene (red represent sodium and green chlorine ions; water molecules are not shown for clarity); (B) axial density profiles (scaled to give the same area under each curve as normalized water density) showing a significantly broader density distribution of the FAM-dsDNA and formation of Na^+^-rich layer at the MXene surface. The *z*-axis origin is set at the top of MXene surface.

**Fig. 3:**
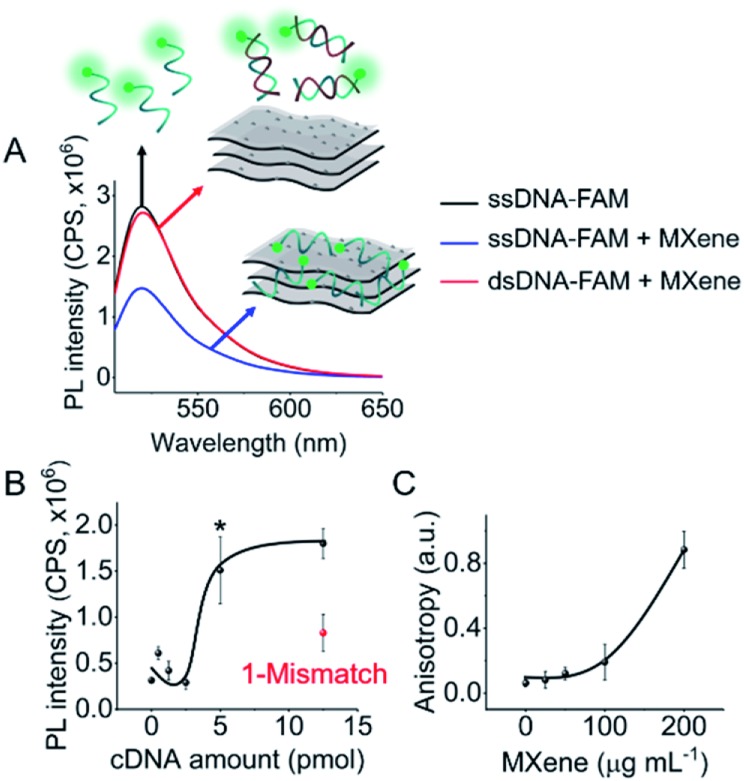
DNA–MXene interaction assessed by fluorescence spectroscopy and anisotropy measurements: (A) fluorescence spectra of FAM-ssDNA, FAM-ssDNA + MXene and FAM-dsDNA + MXene; (B) fluorescence intensity (*λ*_ex_ = 490 nm; *λ*_em_ = 520 nm) *versus* cDNA amount; and (C) anisotropy *versus* MXene concentration. *p < 0.05.

DNA–MXene interaction was assessed with fluorescence spectroscopy by firstly incubating the MXene material with FAM-ssDNA (also denoted as ssDNA). The sequences used in this work, which correspond to an apolipoprotein-E-encoding DNA fragment,^13^ are illustrated in Fig. S8.† FRET is envisioned to take place due to the proximity of the fluorophore, 6-carboxyfluorescein (FAM), covalently bound to one end of ssDNA, to the surface of the material, leading to fluorescence quenching. Fig. S9† shows the spectral overlap between the broad absorption spectrum of Ti_3_C_2_T_*x*_ and the absorption and emission peaks of the FAM dye. Fig. 3A shows that for ssDNA–MXene, the fluorescence of FAM decreased by *ca.* 48% and, in the case of dsDNA, negligible difference can be seen compared to the spectrum of FAM alone, agreeing with the foreseen interaction illustrated in Scheme 1. Further evidence was also provided by MD simulations. In the case of ssDNA, the DNA strand stayed in close contact with MXene surface during 200 ns long MD simulations, as indicated by a broad density maximum of DNA 5.8–9.6 Å (estimated as an interquartile range; median 7.7 Å) from the surface when simulated without the fluorophore, and 7.2–12.6 Å (median 9.9 Å) with the FAM-labeled ssDNA (Fig. 2A and B and S12†). On the other hand, dsDNA and FAM-dsDNA were more distant from the surface with a median of 12.3 Å (interquartile range 8.5–16.1 Å) and 13.2 Å (interquartile range 9.2–17.4 Å), respectively, which could be attributed to a lower interaction of dsDNA to the surface compared to ssDNA. In the case of ssDNA labeled by FAM, the fluorophore interacted mainly with the surface, whereas in the case of dsDNA the FAM not only interacted with the surface but also with the end of the double helix by stacking. These observations can explain the quenched fluorescence of ssDNA which has also been evidenced in recent works,^37^,^58^ and can be assigned to resonant electron transfer given the spectral overlap and the low-range distance. But given the low distance (≤10 Å), the question of whether Dexter energy transfer has a role also arises. Other contributions can be playing a role too such as metal damping, providing additional non-radiative decay of FAM's excited state.^59^ Elucidating the specific mechanisms responsible for the quenching phenomenon observed here is beyond the scope of this work. Inner filter effects can also have implications in the attenuation of fluorescence due to the absorption of light at both excitation and emission wavelengths by the MXene. We however kept the concentration of these absorbing species constant throughout most of the experiments, thus the correction of such effects would not impact the observed trends.

We then proceeded to test different amounts of complementary DNA (cDNA) for the dsDNA + MXene incubation, to assess whether a correlation between fluorescence intensity and cDNA amount was feasible. Fig. 3B shows the nonlinear response of the system. Repeated measurements systematically showed statistically significant (*p* < 0.05) fluorescence changes when cDNA was equal or above 5 pmol, *i.e.* when the ratio of ssDNA to cDNA was at least 1 to 5. Thus, unequivocal detection of 5 pmol of a complementary DNA sequence could be attained with this system. Interestingly, one mismatch gave *ca.* 50% less response than the fully complementary one, suggesting that this platform has potential for sequence-specific discrimination. Even though this needs to be evaluated further with a variety of mismatched sequences, it is relevant to mention that single-base discrimination with other layered/2D materials such as graphene oxide is generally very low, *e.g. ca.* 70–80% of the response obtained with fully complementary strand.^13^,^60^

As a confirmatory tool, we performed fluorescence anisotropy measurements, as these are commonly used to probe biomolecular interactions and affinities.^61^ By placing the fluorescent signal on the smaller FAM molecule binding to the much larger material flakes, substantial changes in anisotropy can be monitored as this binding will significantly decrease the rotational diffusion of the fluorophore. The fluorescence anisotropy of free FAM-ssDNA and the ssDNA–MXene complex was appreciably different: 0.06 and 0.12, respectively (MXene concentration of 50 μg mL^–1^), suggesting that an interaction is taking place.^62^Fig. 3C shows the increase of anisotropy as a function of MXene concentration. Fig. S10† shows the effect of different MXene concentrations on fluorescence intensity and calibration. The fluorescence changes were relevant from 50 μg mL^–1^ on, plateauing after 100 μg mL^–1^ (Fig. S10A†), whereas for calibration we found 50 μg mL^–1^ to be the optimum value with the highest sensitivity (Fig. S10B†). Higher MXene concentration in the media showed a marked decrease in the recovery of fluorescence by increasing cDNA amounts, which can be associated with the high absorbance of the material at these wavelengths. The concentration of MXene was kept constant at 50 μg mL^–1^ throughout subsequent experiments).

**Fig. 4:**
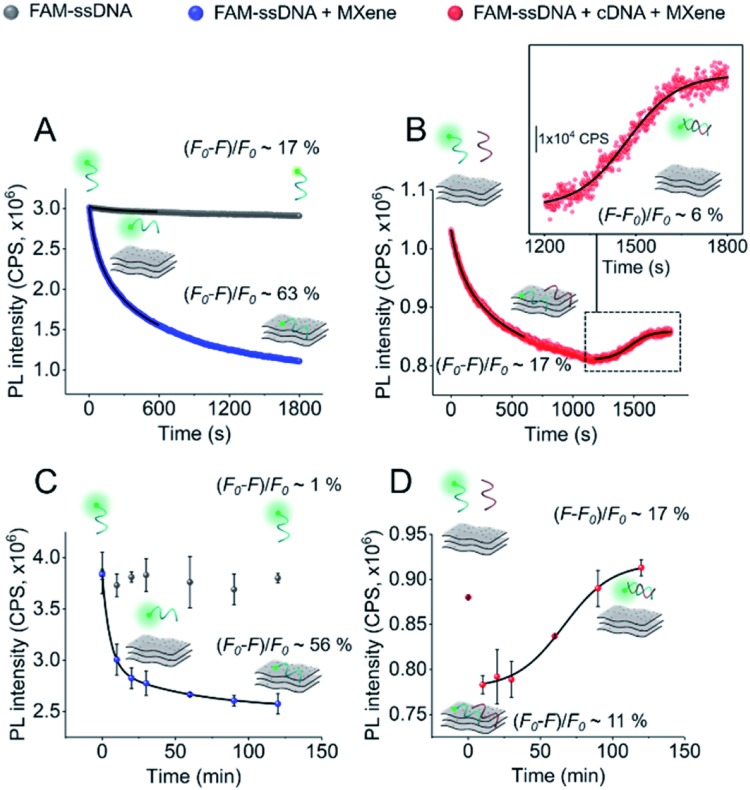
Kinetic profiling of DNA–MXene interaction: (A and B) real-time fluorescence kinetics (step: 1 s; *λ*_ex_ = 490 nm; *λ*_em_ = 520 nm); and (C and D) single-point measurements performed by incubating the samples (protected from light) and taking aliquots for measurements at 0, 10, 20, 30, 60, 90 and 120 min (*λ*_ex_ = 490 nm; *λ*_em_ = 520 nm). (A and C) FAM-ssDNA and FAM-ssDNA (50 nM) + MXene (50 μg mL^–1^) with their respective fluorescence intensity changes ((*F*_0_ – *F*)/*F*_0_, where *F*_0_ is the intensity recorded at 0 s and *F* is the intensity recorded at 1800 s or 120 min). The fluorescence decays were fitted with double exponential functions. (B and D) FAM-ssDNA (50 nM) + cDNA (50 nM) + MXene displaying fluorescence intensity changes from 0–600 s for adsorption and 1200–1800 s or 10–120 min for desorption ((*F* – *F*_0_)/*F*, where *F* is the intensity recorded at 1800 s or 120 min and *F*_0_ is the intensity recorded at 1200 s or 10 min). Inlet of (B) shows the region of fluorescence increase (1200–1800 s) fitted with a sigmoidal (Boltzmann) fit. The increase in fluorescence in plot (D) (10–120 min) was also fitted with a sigmoidal (Boltzmann) function. PL intensity was normalized in (A and B) to FAM-ssDNA's at time zero.

In order to gain more information about the system, we carried out a kinetic assessment of DNA–MXene binding (Fig. 4). This kind of experiments has been typically undertaken in previous reports as follows: (1) ssDNA–fluorophore is incubated with the material and fluorescence changes are monitored either in time-resolved measurements for the calculation of quenching efficiencies/mechanisms or in larger time scales to withdraw information on binding kinetics; (2) the ssDNA–material complex is isolated and purified and then exposed to cDNA in order to record the increase in fluorescence as a function of time, due to desorption of ssDNA off the surface to hybridize with cDNA. The second phase of this experimental assessment requires several centrifugation/washing cycles and often involves the use of centrifugal filtration devices with cut-off molecular weight specifically selected to entrap DNA–material complexes and to get rid of unbound DNA strands. The MXene prepared in this work underwent re-stacking after subsequent centrifugation/washing cycles. The resulting cake could not be redispersed in the ionic strength working conditions, making it difficult to separate ssDNA–MXene from unbound/free ssDNA. This led us to assess the binding kinetics by a distinct approach, *i.e.* on one hand, ssDNA + MXene incubation was carried out and, on the other hand, a three-component system in one-pot reaction was used to assess desorption kinetics: ssDNA + cDNA + MXene. Fig. 4A shows the kinetic profiling of free FAM-ssDNA and FAM-ssDNA incubated with MXene over the course of 30 min. The measurement was done in real-time mode, *i.e.* fluorescence emission was recorded every second while the sample was constantly irradiated at 490 nm. This approach needs to consider the unavoidable photobleaching of the fluorophore, thus the loss of fluorescence, registered in terms of fluorescence changes ((*F*_0_ – *F*)/*F*_0_), was *ca.* 17% for the FAM system alone, and *ca.* 63% in the presence of the material.

Fig. 4B shows that in the three-component system, the desorption of FAM-ssDNA takes place after 20 min of reaction time, resulting in a fluorescence recovery of *ca.* 6%. Fig. 4B and C show the kinetic profiling experiments carried out in a longer time scale (up to 2 h) in a single-point fashion so that the fluorescence changes remained minimally affected by photobleaching. As a result of minimizing such contribution, the fluorescence decrease and subsequent recovery were lower and higher, respectively. The real-time adsorption/desorption profile (Fig. 4B and D) can be conceptually explained by the notion that the reaction between nucleic acids and MXene is kinetically favorable, however DNA–DNA hybridization subsequently governs the system as a thermodynamically-controlled reaction. The latter induces the partial desorption of both DNA sequences off the surface of the material, leading to the recovery of fluorescence.

**Scheme 2:**
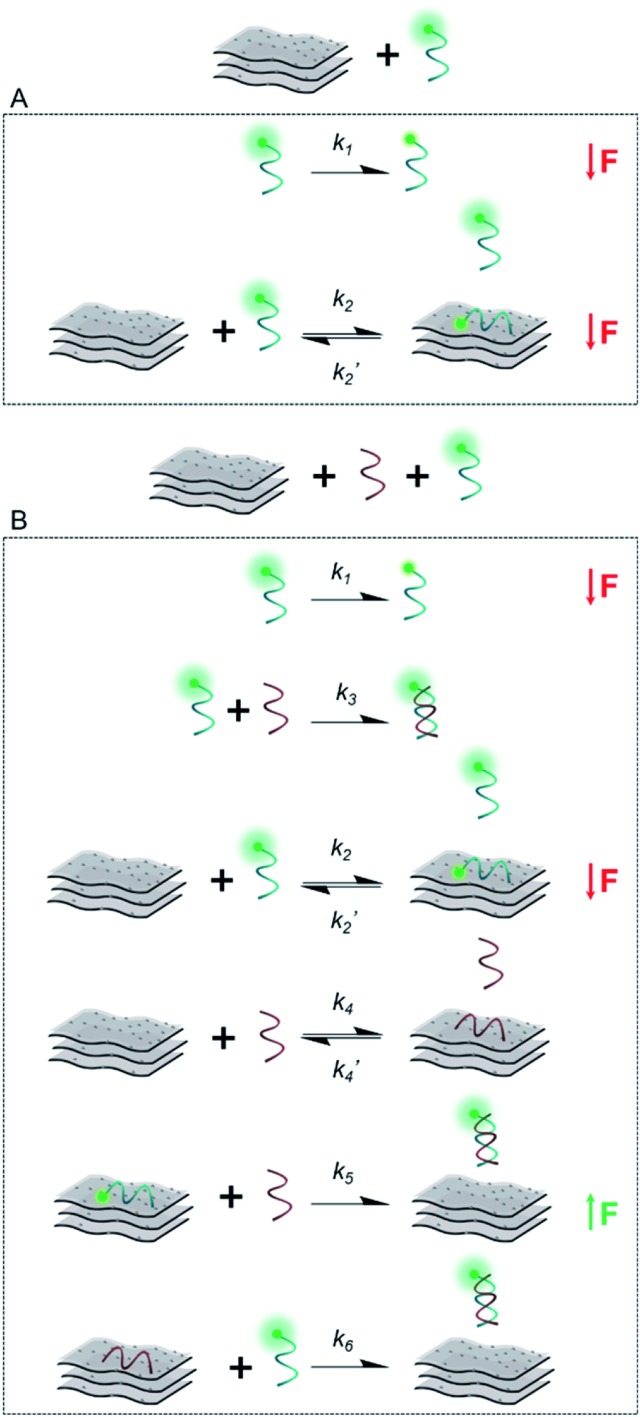
Simplified representation of the plausible kinetic processes taking place between (A) the material and FAM-ssDNA and (B) the material, FAM-ssDNA and cDNA (the two oligonucleotides in equimolar ratio) and their effect on fluorescence intensity.

Scheme 2 illustrates the plausible kinetic processes taking place in the two- and three-component systems. The processes are: photobleaching of the FAM dye, governed by *k*_1_; FAM-ssDNA adsorption onto MXene (*k*_2_); DNA hybridization (*k*_3_); adsorption of cDNA onto MXene (*k*_4_); desorption of FAM-ssDNA off the MXene surface, induced by hybridization with cDNA (*k*_5_); desorption of cDNA off the MXene surface, induced by hybridization with FAM-ssDNA (*k*_6_).

This unique behavior of fluorescence decrease followed by fluorescence recovery in one-pot reaction has not been reported with other materials, to the best of our knowledge. Reference and comparable materials, *e.g.* graphene oxide and TiO_2_, have been tested by our group. Graphene oxide adsorbs DNA mainly *via* the nucleobases by hydrogen bonding and π–π stacking interactions,^63^,^64^ while TiO_2_ nanoparticles are known to bind DNA mainly *via* the phosphate backbone at pH 7.4, and also interacting with the bases to a lesser extent.^65^ The kinetic profiling does not exhibit in either case a real-time adsorption/desorption behavior under the same reaction conditions used for MXene. Given the TiO_2_-decorated features on the MXene surface, we show the adsorption/desorption profile of DNA onto TiO_2_ nanoparticles in Fig. S11,† where there is no recovery of fluorescence after 30 min. In the case of MXene the MD simulation indicated almost no stacking interaction of DNA with the surface and, rarely, hydrogen bond formation between the DNA and MXene molecules. Additionally, ions bound at the surface formed several ion bridges with the DNA molecule (Fig. S13†). as-prepared. The MXene dispersion displayed negative zeta potential (*ca.* –30 mV), which further suggests that the interaction with DNA most likely takes place *via* ion bridges.^58^

MXenes constitute a complex scenario brought by their multielement composition, richness in surface ligands and TiO_2_ nano-sized species, making it challenging to intuitively elucidate the binding mechanism with nucleic acids. The entrapment/release behavior particularly suggests that the interaction is weaker than in the other systems tested,^58^*i.e.* graphene oxide and TiO_2_, and thus the displacement occurs spontaneously after a short time of exposure to the complementary sequence. Why such spontaneous entrapment/displacement behavior occurs with MXene in this time frame and not with a chemically-related system such as TiO_2_ remains an open question. Further studies will be carried out to investigate the variables affecting such phenomenon, as well as the implications in polymorphism differentiation.

## Conclusions

We have investigated the interaction between DNA and MXenes *via* fluorescence spectroscopy and molecular dynamics simulations. The multifaceted features of MXenes make up a complex system that is not only capable of catching nucleic acids *via* ion bridges, but due to the proximity (≤10 Å) of such interaction, the fluorescence of dye-labeled DNA can be quenched, offering a potential biosensing platform with a relevant degree of mismatch discrimination. The weak nature of the interaction allows for a kinetically-dynamic system with interesting adsorption/desorption features, making MXenes unique among other layered/2D materials. These early findings reveal the versatility and promising properties of this family of materials in the biomedical field, specifically their potential use in controlled delivery of nucleic acids and advanced biosensing systems.

## Conflicts of interest

There are no conflicts of interest to declare.

## Supplementary Material

Supplementary information
