# Supplementary material for: Interaction of single- and double-stranded DNA with multilayer MXene by fluorescence spectroscopy and molecular dynamics simulations
Source: Chem Sci. 2019 Sep 23;10(43):10010–7. doi: 10.1039/c9sc03049b (PMC6979399; doi:10.1039/c9sc03049b)
Supplement: Supplementary file 1 [file SC-010-C9SC03049B-s001.pdf]

Electronic Supplementary Material (ESI) for Chemical Science.
This journal is © The Royal Society of Chemistry 2019

Interaction of single- and double-stranded DNA with multilayer MXene by fluorescence spectroscopy and molecular dynamics simulations

C. Lorena Manzanares-Palenzuela,[a] Amir M. Pourrahimi,[a] J.Gonzalez-Julian,[b] Zdenek Sofer,[a] Martin Pykal,[c] Michal Otyepka[c] and Martin Pumera[a]*

[a] Center for Advanced Functional Nanorobots, Department of Inorganic Chemistry, University of Chemistry and Technology Prague, Technická 5, 166 28, Prague 6 (Czech Republic)
[b] Forschungszentrum Jülich GmbH, Institute of Energy and Climate Research, Materials Synthesis and Processing (IEK-1), 52425 Jülich, Germany
[c] Regional Centre of Advanced Technologies and Materials, Palacký University Olomouc, Šlechtitelů 27, 771 46 Olomouc (Czech Republic)

*E-mail: martin.pumera@vscht.cz

Contents

Experimental section 3

Figure S1. Typical SEM images of MXene $Ti_3C_2T_x$ obtained by HF etching, followed by water intercalation *via* ultrasonication: Top view of multilayer flakes (above) and large etched accordion-like structures (below) retrieved from main dispersion. Blue and white scale bars are 1 μm and 500 nm, respectively. 4

Figure S2. Representative AFM images of MXene flakes retrieved from the supernatant of the main dispersion and their corresponding height/width profiles. 5

Figure S3. A, B) TEM and C) high-resolution TEM images of MXene; D) selected area electron diffraction (SAED) pattern. 6

Figure S4. XRD pattern of MAX phase and MXene. 7

Figure S5. High-resolution XPS of the O 1s region for intercalated Mxene. 8

Figure S6. Absorbance spectrum of the stable MXene multilayer flakes dispersed in Tris buffer. 9

Figure S7. NLDFT pore volume and cumulative pore volume distribution for A) MAX phase precursor and B) resulting MXene powder after HF-etching. 10

Figure S8. DNA structures used modelled by Mfold software (http://unafold.rna.albany.edu/). 11

Figure S9. Spectral overlap of MXene absorption, FAM absorption and emission. 12

Figure S10. Effect of MXene concentration on A) fluorescence changes of FAM-ssDNA (F_0 and F stand for the intensity values of the fluorophore in the absence and presence of material) and B) intensity recorded with increasing cDNA amounts. 13

Figure S11. Real-time kinetic profiling of TiO_2 (20 nm nanoparticles, P25 Degussa) 50 μg mL^{-1} incubated with FAM-ssDNA 50 nM and with FAM-ssDNA and cDNA at equimolar ratio. 14

Figure S12. A) Structures taken from MD simulation showing the typical binding pattern of ssDNA (left) and dsDNA (right) on MXene. Ions further than 15 Å from the DNA are shown as semi-transparent spheres (red represent sodium and green chlorine ions; water molecules are not shown for clarity). B) Axial density profiles (scaled to give the same area under each curve as normalized water density) showing a significantly broader density distribution of the dsDNA and formation of Na+-rich layer at the MXene surface. The z-axis origin is set at the top of MXene surface 15

Figure S13. The time evolution of the number of A) nucleobases in stacking contacts (i.e., nucleobases that are oriented 90±15º to the surface normal and within a distance of 5.5 Å from the surface), B) hydrogen bonds (calculated using the *g_hbond* tool of GROMACS) and C) ion bridges (simultaneous interaction of MXene-ion-DNA, both within a distance of 3.5 Å) between the ssDNA (shown on the left panels) and dsDNA (shown on the right panels) and MXene during the course of molecular dynamics simulation. 16

References 17

Experimental section

Synthesis of Ti_3AlC_2. Elemental powders of Ti, Al (>99.5%, Alfa Aesar, -325 mesh) and graphite (Alfa Aesar, 2 μm) were mixed in the stoichiometric molar ratio of 3:1.2:2. KBr (Alfa Aesar) in 1:1 weight ratio was added to the resulting mix. The mixing was done with ethanol and zirconia milling balls (Φ 5mm) for 24 hours in a multidirectional mixer (Turbula, WAB, Switzerland). The dried powder was uni-axially pressed in a steel die under a pressure of 200 MPa. The pressed powders were further encapsulated with KBr and placed in a KBr salt bed in an alumina crucible covered with an alumina lid. The reaction mixture was heated to 1300°C at a rate of 5°C/min with a dwell time of 1 hr at 1300°C. After cooling, the Ti_3AlC_2 was obtained by dissolving the reaction mixture in water. The obtained Ti_3AlC_2 powders were washed in hot water to eliminate the residual KBr salt content. Finally, the Ti_3AlC_2 powders were rinsed in ethanol to avoid agglomeration and dried at 70°C in an oven.

Ti_3AlC_2 exfoliation. The Ti_3AlC_2 phase was grinded and friction with particle size below 0.1 mm was used for exfoliation. The exfoliation of Ti_3AlC_2 was performed using 40% wt. HF acid at room temperature. 5 g of Ti_3AlC_2 was added in 250 mL of 40% HF in 1L PP Erlenmeyer flask and ultrasonicated for 30 minutes. Subsequently, the reaction mixture was stirred at room temperature for 7 days. The exfoliated formed Ti_3C_2Tx MXene was separated by suction filtration (using PTFE filtration apparatus), repeatedly washed with water and dried in vacuum oven (24 hours / 50 °C). The powder was resuspended in water and let to sediment to retrieve the fine particles in the supernatant.

Characterization. A field-emission scanning electron microscope (SEM; TESCAN MAIA 3) coupled with energy dispersive spectrometer (EDS) (Oxford Instruments, UK) was used to assess the morphology and elemental mapping. X-ray Powder Diffraction (XRD) was carried out by Bruker D8 Discoverer (Bruker, Germany) powder diffractometer with a Cu Kα source. The chemical composition of the etched MXene was assessed with X-ray photoelectron spectroscopy (XPS) by performing high-resolution scans of the Ti 2p and O 1s regions. The XPS system consisted of an XR-50-MF X-ray source with a μ-Focus monochromator and Phoibos 150 2D CCD hemispherical detector (SPECS, Germany). Atomic Force Microscopy (AFM) was performed with Flex-Axiom “Nanosurf” under contact mode in dry state. For absorbance measurements, a Multiskan Sky spectrophotometer (Thermo Scientific) was used. Brunauer–Emmett–Teller (BET) nitrogen adsorption/desorption measurements were made using NOVAtouch 2200 (Quantachrome, USA) at 77 K. The samples were heated to 200 °C prior to each measurement to remove all adsorbed water. Raman spectroscopy was carried out with an inVia Raman microscope (Renishaw, England) in backscattering geometry with a charge-coupled device detector. DPSS laser (532 nm, 50 mW) with an applied power of 5 mW and a 50× magnification objective was used for the measurement. High-resolution TEM (HR-TEM) was done with an EFTEM JEOL 2200 FS microscope (JEOL, Japan). A 200 keV acceleration voltage was used for the measurements.

DNA binding studies. An aqueous MXene dispersion and FAM-ssDNA solution were incubated to a final concentration of 50 μg mL^{-1} and 50 nM (unless otherwise specified) respectively, in Tris buffer (50 mM Tris-HCl, 100 mM NaCl, 10 mM $MgCl_2$, pH 7.4). Incubation time was set to 1 h, before recording photoluminescence (PL) (fluorescence) spectra and end-point measurements. For MXene + dsDNA experiments, firstly FAM-ssDNA and cDNA were incubated at 50 nM and 1 to 50 nM, respectively, in Tris buffer for 1 h. The formed hybrids were transferred to aqueous MXene dispersion 50 μg mL^{-1}, followed by another 1-h incubation. Kinetic measurements were carried out by incubating MXene + FAM-ssDNA and MXene + FAM-ssDNA + cDNA (50 nM) as described above and recording the fluorescence intensity over time in real-time or in single-point measurements. PL measurements were recorded at an excitation wavelength of 490 nm using Fluorolog Extreme (Horiba, France) equipped with a Xe lamp (450 W) and a double excitation monochromator. A monochromator iHR320 with a thermoelectrically cooled PMT detector was used for the measurement of emission spectra. Fluorescence anisotropy was recorded using L-format. All experiments were done at room temperature keeping the samples protected from light.

Simulation setup. Molecular dynamics (MD) simulations were performed using Gromacs 4.6.3 program package.[1] The used DNA structures were reduced from 24 nucleotides to 12 nucleotides (while preserving the original sequence) in order to reduce computational demands. DNA sequences were generated using the make-na server (http://structure.usc.edu/make-na). DNA was simulated both with and without the fluorescent FAM probe. The parm10 forcefield with the OL15 refinement[2] for DNA has been used for the DNA. The periodic $Ti_3C_2T_x$ MXene (box size 90 x 90 x 80 Å) was simulated using the parameters published by Xu et al.[3] MXene was functionalized with -F, -OH, -O groups with the ratio of 1.02 : 0.12 : 0.79, giving the final formula: $Ti_3C_2OH_{1.02}F_{1.02}O_{0.79}$.[4] Three initial configurations were considered; ssDNA and dsDNA structure (oriented in an upright or in parallel position with respect to the surface) was placed 20 Å above the MXene and solvated using SPC/E water model.[5] Na^+ and Cl^- ions[6] at a 150 mM concentration was added to neutralize the system. Alternatively, the behaviour of DNA on MXene was studied also in KCl[6] solution and similar behavior was observed. The surface of the MXene was kept restrained during all simulations. At first, the system was minimized and thermalized using a V-rescale thermostat[7] and Berendsen barostat[8] (from 10 to 300 K and p = 1 bar), whereby the heavy atoms of DNA were kept restrained. Subsequently, 20 ns was used for the NpT equilibration at the final temperature and then switched to the NVT ensemble for the production run; in total 200 ns for each MX···DNA configuration. Non-bonded interactions were truncated at 10 Å. The electrostatics was handled using particle-mesh Ewald (PME) with a real-space cut-off 10 Å. Periodic boundary conditions were used in all three dimensions. The equations of motion were integrated with a 2 fs time step. Bonds involving hydrogens were constrained by LINCS algorithm.[9]

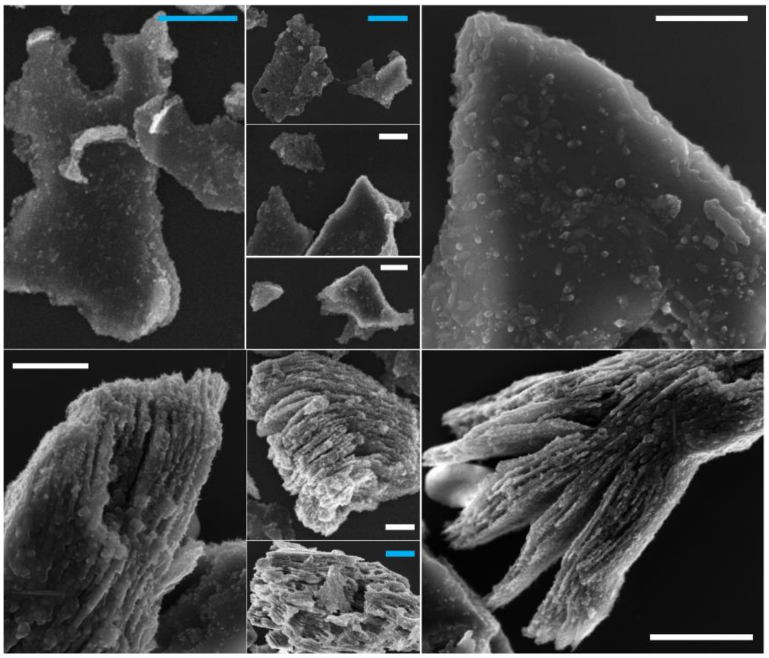

Figure S1. Typical SEM images of MXene $Ti_3C_2T_x$ obtained by HF etching, followed by water intercalation *via* ultrasonication: Top view of multilayer flakes (above) and large etched accordion-like structures (below) retrieved from main dispersion. Blue and white scale bars are 1 μm and 500 nm, respectively.

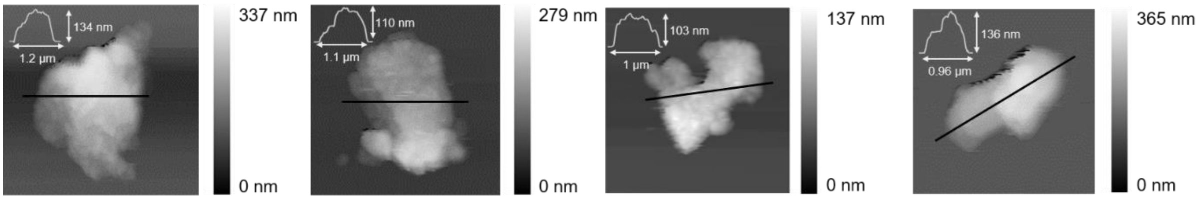

Figure S2. Representative AFM images of MXene flakes retrieved from the supernatant of the main dispersion and their corresponding height/width profiles.

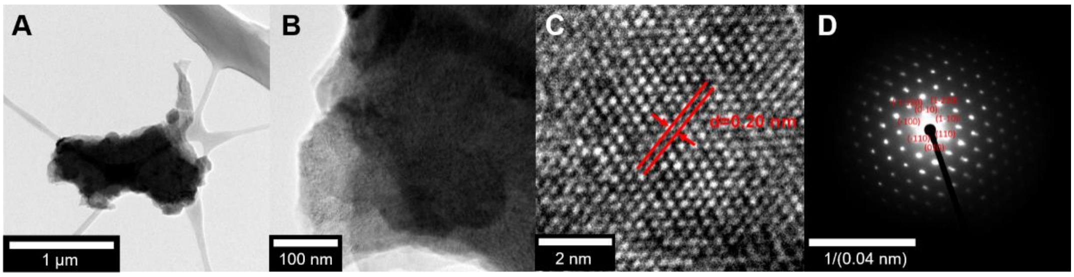

Figure S3. A, B) TEM and C) high-resolution TEM images of MXene; D) selected area electron diffraction (SAED) pattern.

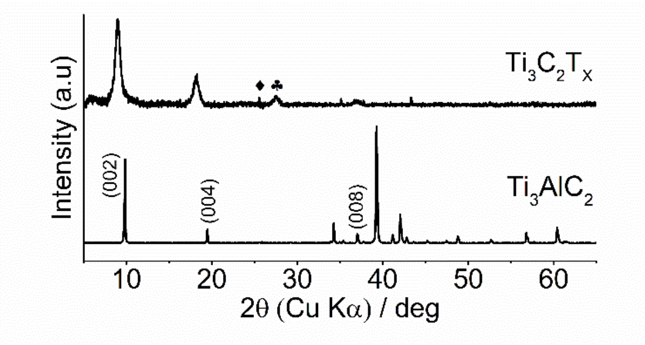

Figure S4. XRD pattern of MAX phase and MXene (♦ anatase, ♣ rutile).

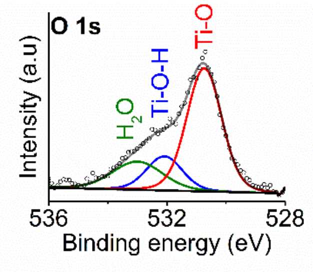

Figure S5. High-resolution XPS of the O 1s region for intercalated MXene.

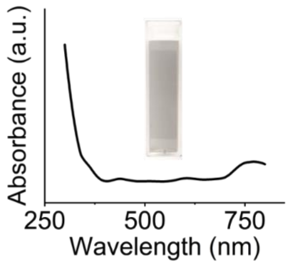

Figure S6. Absorbance spectrum of the stable MXene multilayer flakes dispersed in Tris buffer.

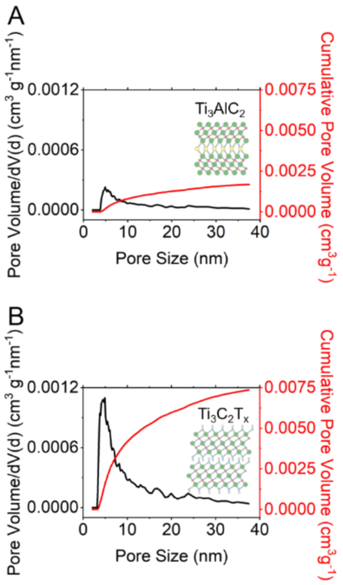

Figure S7. NLDFT pore volume and cumulative pore volume distribution for A) MAX phase precursor and B) resulting MXene powder after HF-etching.

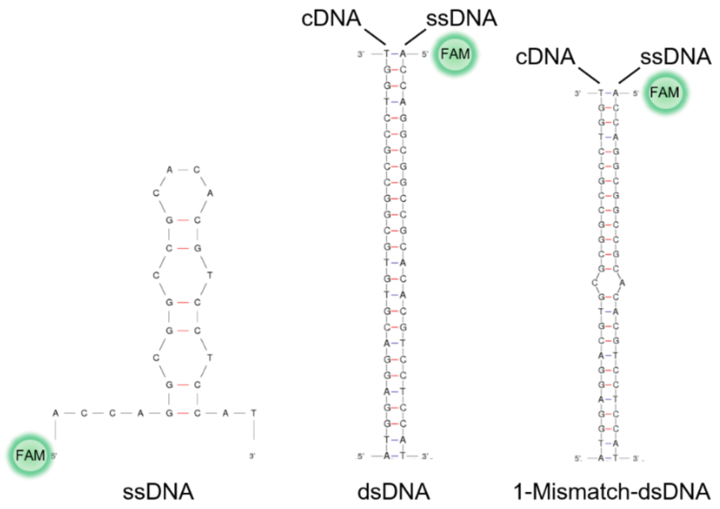

Figure S8. DNA structures used modelled by Mfold software (http://unafold.rna.albany.edu/)

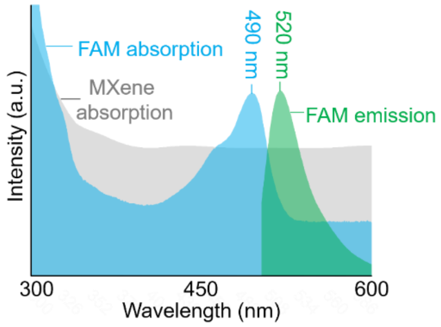

Figure S9. Spectral overlap of MXene absorption, FAM absorption and emission.

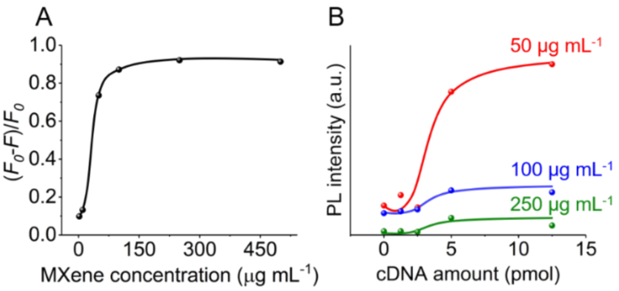

Figure S10. Effect of MXene concentration on A) fluorescence changes of FAM-ssDNA (F_0 and F stand for the intensity values of the fluorophore in the absence and presence of material) and B) intensity recorded with increasing cDNA amounts.

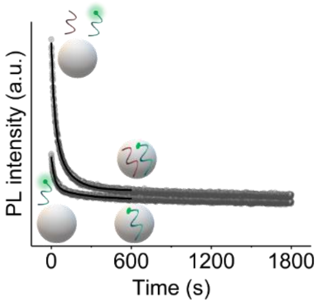

Figure S11. Real-time kinetic profiling of TiO_2 (20 nm nanoparticles, P25 Degussa) 50 µg mL^{-1} incubated with FAM-ssDNA 50 nM and with FAM-ssDNA and cDNA at equimolar ratio.

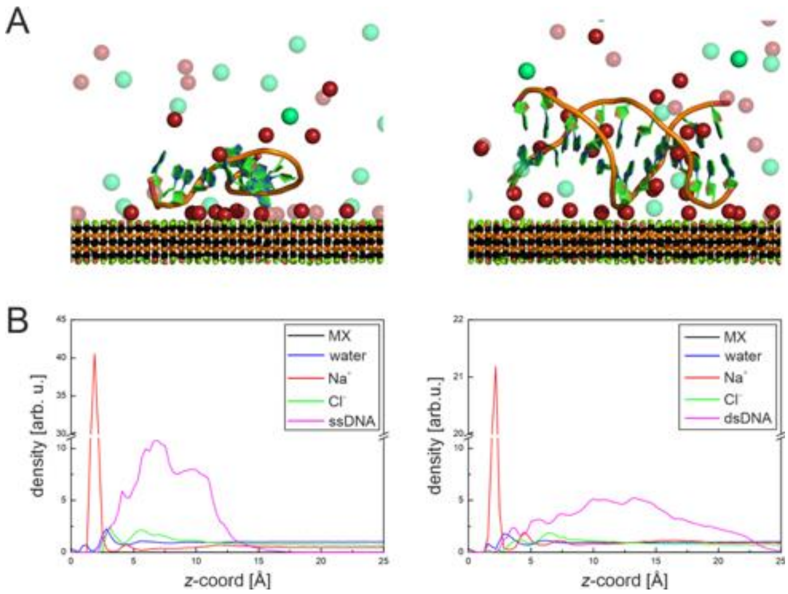

Figure S12. A) Structures taken from MD simulation showing the typical binding pattern of ssDNA (left) and dsDNA (right) on MXene. Ions further than 15 Å from the DNA are shown as semi-transparent spheres (red represent sodium and green chlorine ions; water molecules are not shown for clarity). B) Axial density profiles (scaled to give the same area under each curve as normalized water density) showing a significantly broader density distribution of the dsDNA and formation of Na^{+}-rich layer at the MXene surface. The z-axis origin is set at the top of MXene surface.

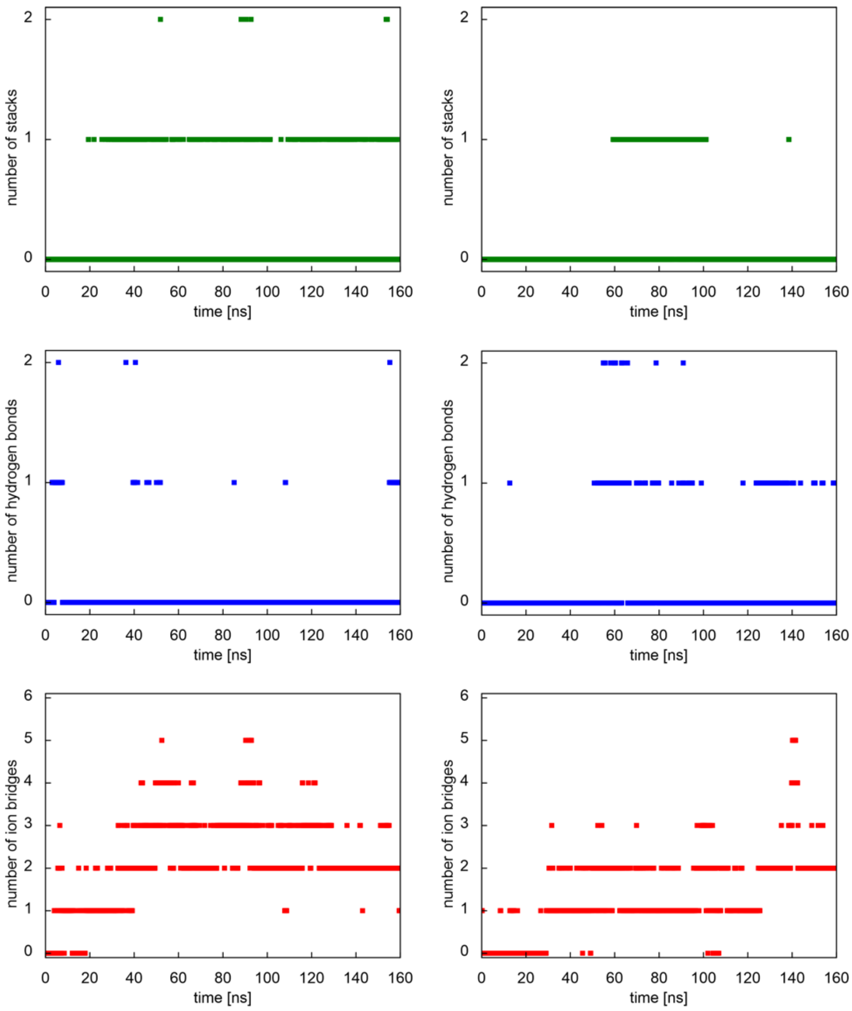

Figure S13. The time evolution of the number of A) nucleobases in stacking contacts (i.e., nucleobases that are oriented 90±15º to the surface normal and within a distance of 5.5 Å from the surface), B) hydrogen bonds (calculated using the *g_hbond* tool of GROMACS) and C) ion bridges (simultaneous interaction of MXene-ion-DNA, both within a distance of 3.5 Å) between the ssDNA (shown on the left panels) and dsDNA (shown on the right panels) and MXene during the course of molecular dynamics simulation.

References

[1] D. V. D. Spoel, E. Lindahl, B. Hess, G. Groenhof, A. E. Mark, H. J. C. Berendsen, *J. Comput. Chem.* **2005**, *26*, 1701-1718.

[2] M. Zgarbová, J. Šponer, M. Otyepka, T. E. Cheatham III, R. Galindo-Murillo, P. Jurečka, *J. Chem. Theory Comput.* **2015**, *11*, 5723–5736.

[3] K. Xu, Z. Lin, C. Merlet, P. L. Taberna, L. Miao, J. Jiang, P. Simon *ChemSusChem* **2018**, *11*, 1892-1899.

[4] M. A. Hope, A. C. Forse, K. J. Griffith, M. R. Lukatskaya, M. Ghidiu, Y. Gogotsi, C. P. Grey, *Phys. Chem. Chem. Phys.* **2016**,*18*, 5099-5102.

[5] H. J. C. Berendsen, J. R. Grigera, T. P. Straatsma, *J. Phys. Chem.* **1987**, *91*, 6269-6271.

[6] I. S. Joung, T. E. Cheatham III, *J. Phys. Chem. B* **2008**, *112*, 9020-9041.

[7] G. Bussi, D. Donadino, M. Parrinello, *J. Chem. Phys.* **2007**, *126*, 014101.

[8] H. J. C. Berendsen, J. P. M. Postma, W. F. van Gunsteren, A. DiNola, J. R. Haak, J. Chem. Phys. 1984, 81, 3684-3690.

[9] B. Hess, H. J. C. Berendsen, J. G. E. M. Fraaije, *J. Comput. Chem.* **1997**, *18*, 1463-1472.

[10] P. Söderhjelm, G. A. Tribello, M. Parrinello *PNAS* **2012**, *109*, 5170.
